# Results of Arthroscopic Repair of Peripheral Triangular Fibrocartilage Complex Tear With Exploration of Dorsal Sensory Branch of Ulnar Nerve

**DOI:** 10.2174/1874325001711010525

**Published:** 2017-05-31

**Authors:** Alvin Chao-Yu Chen, Chun-Jui Weng, Chih-Hao Chiu, Shih-Sheng Chang, Chun-Ying Cheng, Yi-Sheng Chan

**Affiliations:** Bone and Joint Research Center, Department of Orthopaedic Surgery, Chang Gung Memorial Hospital-Linkou & University College of Medicine; Taiwan, Republic of China

**Keywords:** Arthroscopy, Dorsal sensory branch of ulnar nerve (DSBUN), Paresthesia, Peripheral tear, Triangular fibrocartilage complex (TFCC), Wrist score

## Abstract

**Background:**

Ulnar-sided approach in arthroscopic triangular fibrocartilage complex (TFCC) repair may jeopardize treatment success by exposing the dorsal sensory branch of ulnar nerve (DSBUN) in risk of injury. We aim to conduct a follow-up assessment of arthroscopic outside-in TFCC repair and efficacy of sensory nerve exploration.

**Methods:**

We conducted a retrospective chart review of 58 patients (59 wrists) who received arthroscopic repair of the peripheral attachment of the TFCC. Ulnar-sided skin incision and exploration of DSBUN were performed before arthroscopy setting. Arthroscopic outside-in repair through pullout suture ligation was performed. Functional survey at 6 months and 1 year postoperatively was based on Mayo Modified Wrist Score (MMWS), and compared to the preoperative assessment. A p-value of less than 0.05 was considered significant as calculated using paired t-test.

**Results:**

Postoperative MMWS averaged 74.32±11.50 at 6 months, and 84.41±9.52 at one year; both showed significant difference as compared to preoperative status. Significant improvement was noted in all 4 individual items except motion retrieval between 6 months and 1 year. Totally, 45 (76%) cases achieved good or excellent results at one year; however, less patients resumed pre-injury activity level when treatment delay was more than 6 months than those treated earlier (41% vs. 57%). Complication included 6 transient paresthesia; 1 anchor migration and 1 distal radioulnar arthrosis. No more nerve complication was found after modification of perineural dissection.

**Conclusion:**

Arthroscopy is effective in obtaining both correct diagnosis and treatment of peripheral TFCC tear. Modified perineural dissection can minimize sensory nerve complications.

## INTRODUCTION

Traumatic disruption of the Triangular Fibrocartilage Complex (TFCC) is a common cause of ulnar-sided wrist pain and is often associated with decreased grip strength and impaired function [1]. The Palmer classification defines traumatic tears involving the ulnar periphery of the TFCC as type 1-B [2]. Arthroscopic repair of peripheral triangular fibrocartilage complex (TFCC) tears is now a commonly accepted method [3, 4]. It provides excellent functional outcome, aided in part by the vascularity of the peripheral TFCC. Owing to the anatomical proximity, the dorsal sensory branch of the ulnar nerve (DSBUN) is at risk in setting the 6U wrist arthroscopy portal [5]. Anatomical variation as well as tissue distortion with wrist traction cautions the surgeons to identify and protect the DSBUN before setting the 6U portal [6]. Currently described repairs of the TFCC require subcutaneous suture knots to be used on the ulnar aspect of the wrist to secure the repair [7, 8].Although surgeons know the risk and are careful when they set the 6U portal and insert needle for suture passing, DSBUN injuries still occur [9, 10]. It has been reported that there is an approximate 50% chance of nerve branch strangulation in passing repair suture [11].

In this study, we introduced a simple and straightforward method to explore the DSBUN and to dissect the surrounding tissue with protection of the nerve throughout the surgical procedure. The functional outcome and complications were meticulously surveyed and reported.

## MATERIALS AND METHODS

A retrospective study to assess distal radio-ulnar joint instability managed by arthroscopic repair of the peripheral attachment of the TFCC from 2004 to 2012 was undertaken in our institution. Institutional review board approval (IRB 201700070B0) was obtained for a review of patients’ records and radiographs. A total of 58 patients (59 wrists) were recruited (Table **1**); the other 8 were excluded due to incomplete data records. Thirty-five male (59%) and 23 female (41%) patients with a mean age of 34.95±10.57 (range, 17 to 57) years were evaluated. All patients were surveyed clinically and radiographically at a range of 1 to 3 months before surgery. Clinical presentation was initiated by traumatic events in 48 of 59 wrists (81%); among them, 11 wrists (19%) were sports related injuries including 8 wrists (14%) in 8 athletes. Among 59 wrists, 24 (41%) were in the left hand, and 35 (59%) in the right. The dominant hand was involved in 42 wrists (71%). According to patients’ records, most common initial complaint was ill-defined pain, which could refer to ulna side of the wrist with an occasional description of a clicking sensation. Pain was also elicited by passively pronating or supinating the wrist while stabilizing the forearm. On clinical evaluation, fovea sign (tenderness close to ulnar styloid) and load shift test (painful piano key sign on ulnar deviation) were the most consistent findings. In most patients, power grip was significantly diminished. A positive magnetic resonance imaging (MRI) scan was obtained in 8 (53%) of 15 referred cases. MRI plus arthrogram was performed for the other 44 wrists in our institution, and showed correlated findings in 40 cases (91%). Arthroscopic findings further confirmed the radiographic diagnoses. All the negative imaging data in 7 (47%) of MRI cases and 4 (9%) of MRI plus arthrogram cases were reconfirmed blindly by a senior radiologist (> 10 years practice); the diagnosis of TFCC tear was directly made during arthroscopy.

### Surgical Procedures

All operations were performed with patients under general anesthesia. A pneumatic tourniquet was routinely used with a mean pressure of 250 mm Hg. Before arthroscopy, the location of 6-U portal was identified and a 1.5-cm longitudinal skin incision was made proximally and distally. Blunt dissection was performed to explore the DSBUN. The sensory nerve was meticulously protected with a vascular loop during the whole course of surgery on the traction tower. By the use of a traction tower (ConMed, Largo, FL), a 2.5-mm 30° arthroscope was introduced into the 3-4 portal by the standard technique. Outflow was placed through the 6-U portal away from the DSBUN, and an accessory portal included the 6-R or 4-5 portal. After investigating the conditions of the TFCC surface, TFCC resilience (trampoline effect) on ballottement was tested [12]. A probe was placed in the 6R portal to detect a peripheral tear, and to evaluate its elasticity (hook test) [13]. The final diagnosis of peripheral TFCC tear was determined by a loss of the normal trampoline effect and displacement of the TFCC distally and radially (Fig. **1A**), and further categorized according to Atzei’s classification system [14].

The peripheral tear was repaired using 2-0 Ethibond sutures (Ethicon, Somerville, NJ, USA) (Fig. **1B**). Pullout suture ligation was performed with suture passing by an outside-in technique [15] through a simple 18-gauze needle (Video clips). Usually 2 to 3 knots were used depending on the tear size and location. In some cases, with a bigger chronic tear, Minitac Ti 2.0 suture anchor (Smith & Nephew, Andover, Massachusetts, USA) was also used to unload the tension of the repaired tissue when residual gap might exist with suture ligation alone.

### Postoperative Care

The patient was placed in a long arm splint postoperatively for 4 weeks. At this point, the wrist is shifted to a short arm splint allowing progressive motion to the wrist for another month. The patients then started a range of motion and grip-strengthening exercises, and was allowed to resume normal activity by 3 months postoperatively.

### Functional Assessment

All the patients had more than one year follow-up with regular functional survey preoperatively, at 6 months and one year postoperatively. The results were graded according to the Mayo Modified Wrist Score (MMWS) [15] and compared with preoperative scores. This increase was considered statistically significant as calculated using a paired t-test for a p-value of less than 0.01.

## RESULTS

Time interval from the beginning of symptoms to surgery averaged around 7.22±8.43 (range, 1 to 36) months. After surgery, all patient had at least one year follow-up with an average of 23.07±15.12 months. Functional survey performed preoperatively 6 months, and 1 year postoperatively, is summarized in Table (**2**). Totally, 45 (76%) cases achieved good or excellent results at one year. The average preoperative wrist score was 51.44±15.57 (range, 10 to 70). Postoperative score averaged 74.32±11.50 (range, 40 to 95) at 6 months, and 84.41±9.52 (range, 60 to 100) at one year. This increase in functional scores was statistically significant at postoperative 6 months, as calculated using a paired t-test for analysis of variance (P < .005); so was the improvement of the individual items. Significant improvement was also found between postoperative 6 months and 1 year in total scores, pain, function and grip strength (P < .001). The increase in motion score was less significant after 6 months (P=0.02). Improvement in pain score was dramatic altering from an average of 11.78±6.35 (range, 0-20) preoperatively to 20.59±2.95 (range, 15 to 25) at 6 months after surgery. All except 2 regained pain-free activity (36 wrist, 61%) or occasionally mild pain (21 wrists, 36%) at one year. The improvement in grip strength was also immediately efficacious from an average score of 8.39±3.4 (range, 0-15) before surgery to 15.34±4.99 (range, 5-25) at 6 months and 19.07±5.21 (range, 10-25) at one year, respectively. However, 35 out of 59 wrists (59%) showed less than 90% of grip strength compared to the other hand at 1 year. About 54% (32 of 59) cases had surgery delay for more than 3 months after injury; 37% (22 of 59) cases, more than 6 months. Half of 58 patients (29 wrists) resumed pre-injury activity level including 6 of 8 athletes (75%) before one year. Lower return rates were observed in the cases with treatment delay of more than 6 months (9 wrists, 41%) than in the cases treated earlier (21 wrists, 57%).

No complications were observed in the immediate postoperative period including wound problems, infection, tendon laceration or neurovascular injury, or during follow-up. However, 6 (75%) of initial 8 cases that underwent DSBUN exploration without perineural tissue preservation (Fig. **2A**) complained paresthesia along dorso-ulnar side of the hand and fingers. All neurologic symptoms resolved at 3- month follow-up. For the following 51 wrists, we modified the method of nerve exploration; DSBUN was isolated with preservation of surrounding vascular cuff (Fig. **2B**). No more neurologic complication was found afterwards.

Anchor migration was found in 1 of 7 cases (14%) with Minitac fixation at 1 month. The patient underwent surgical removal of the anchor at 3 months after primary surgery, and regained pain-free motion. One of the two cases (3%) with moderate to severe motion pain at one year underwent subsequent Sauve-Kapanji surgery because of painful arthrosis in distal radioulnar joint.

## DISCUSSION

The most common mechanism occurs with a fall in an outstretched hand with extension and pronation of the axial-loaded carpus [16]. Peripheral tears of the articular disk are also commonly seen in athletic injuries, which involve rapid twisting of the wrist such as the ulnar-sided loading activities of racket sports and golf. In our series, 11 cases (19%) were sports related injuries; among them, 8 were athletes. Six of the 8 athletes could not recall the traumatic event and delayed diagnosis and treatment of the lesions, which may predispose athletes to both acute and chronic TFCC lesions [17]. Early detection of TFCC lesions is crucial, and basically relies on meticulous examination of those without traumatic recall.

Peripheral tears of TFCC can be addressed by one of the several arthroscopic techniques. Good results have been reported for both inside-out and outside-in techniques. Improvement in pain and function were immediate and dramatic. Reduced grip strength and moderate improvement in motion have been mentioned in many reports [18, 19]. Progressive improvement was noted in our study. Functional scores in total and individual items significantly increased (p<0.01) at 6 months and one year except the motion item. The difference in the improvement of motion between 6-month and one-year surveys was statistically insignificant (p=.02). Slight limitation of motion was noted on supination and dorsiflexion while not compromising daily activities. As to grip strength, the improvement at 6 months and one year were both significant; however, only 41% (24 of 59) cases regained more than 90% of the contralateral hand. Half of all patients did not resume pre-injury activity level including 2 athletes; return rate was lower in the cases with treatment delay for more than 6 months. Less favorable outcome could be due to late management since the average time to surgery was up to 7.22 months. Precise, early diagnosis seemed to afford the better chance for promising outcome.

Possible complications may be related to setting-up of the arm traction, establishment of portals, procedure-specific injuries, and general complications involved in wrist arthroscopy [20]. Neurological injury resulting from arthroscopic procedures was commonly described in the literature [21]; many of them were transient, but complete nerve avulsion and neuroma formation have been reported [22, 23]. Injury to the dorsal sensory branches of the ulnar nerve seems to be among the most frequent neurologic complications [9]. Because of the close anatomic relations and the arborization pattern of DSBUN, it was recommended to make a longitudinal incision in the region of 6U portal [24] and to spread the soft tissues with a fine point hemostat prior to instrument insertion [25]. Common concerns had been raised in previous publications while the techniques to explore the nerve varied. In Frank’s technique report, the nerve was explored after suture passage without mentioning complication rate [26]. A recent case series using ulnar-sided repair reported 10.4% cases of DSBUN neurapraxia even separate incisions were made for nerve protection [8]. Similar complication was also found when we initially performed the surgery. We soon modified the way of nerve dissection. Instead of stripping off the perineural tissue, we explored and isolated DSBUN with surrounding vascular cuff preserved to avoid devascularizing the nerve. Then the nerve was well protected throughout the whole course of surgery. All instrumentation, needle insertion and suture ligament were kept away from the nerve to avoid accidental damage as well as nerve strangulation due to postoperative tissue scarring [10].

Our study is attributed to all the limitations of retrospective design, relying on medical records and operative notes for data collection. The excluded cases owing to incomplete records may probably affect the outcome assessment. Our sample size was moderate with wide variability in the age and heterogeneity in disease chronicity; subdividing the trauma mechanism was not allowed. Finally, lack of “second look” arthroscopic evaluation could not provide direct evidence of tissue healing of the repaired TFCC.

## CONCLUSION

Triangular fibrocartilage complex injury is a common etiology of ulnar-sided wrist pain following fall accident and sports injury. Arthroscopy is effective in obtaining both correct diagnosis and treatment of peripheral TFCC tear, which cannot always be clearly diagnosed with preoperative imaging. Arthroscopic suture repair using outside-in technique has been shown encouraging results. Less favorable outcome as to resuming pre-injury level and grip strength recovery may be attributed to late detection and management. Through a longitudinal skin incision and gentle tissue dissection, DSBUN as well as the perineural circulation can be meticulously preserved to minimize the neurological complications.

## Figures and Tables

**Fig. (1A):**
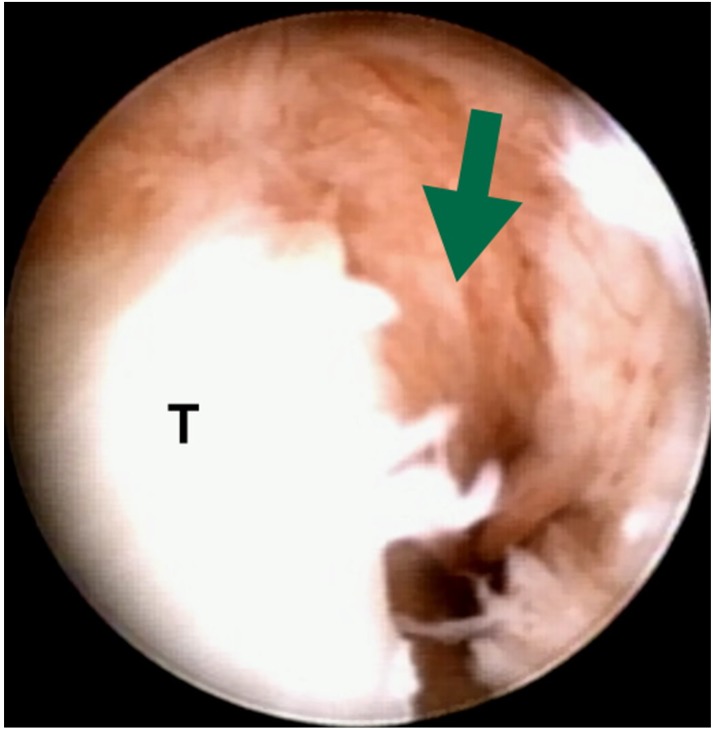
The arrow indicates the peripheral tear of TFCC (T).

**Fig. (1B):**
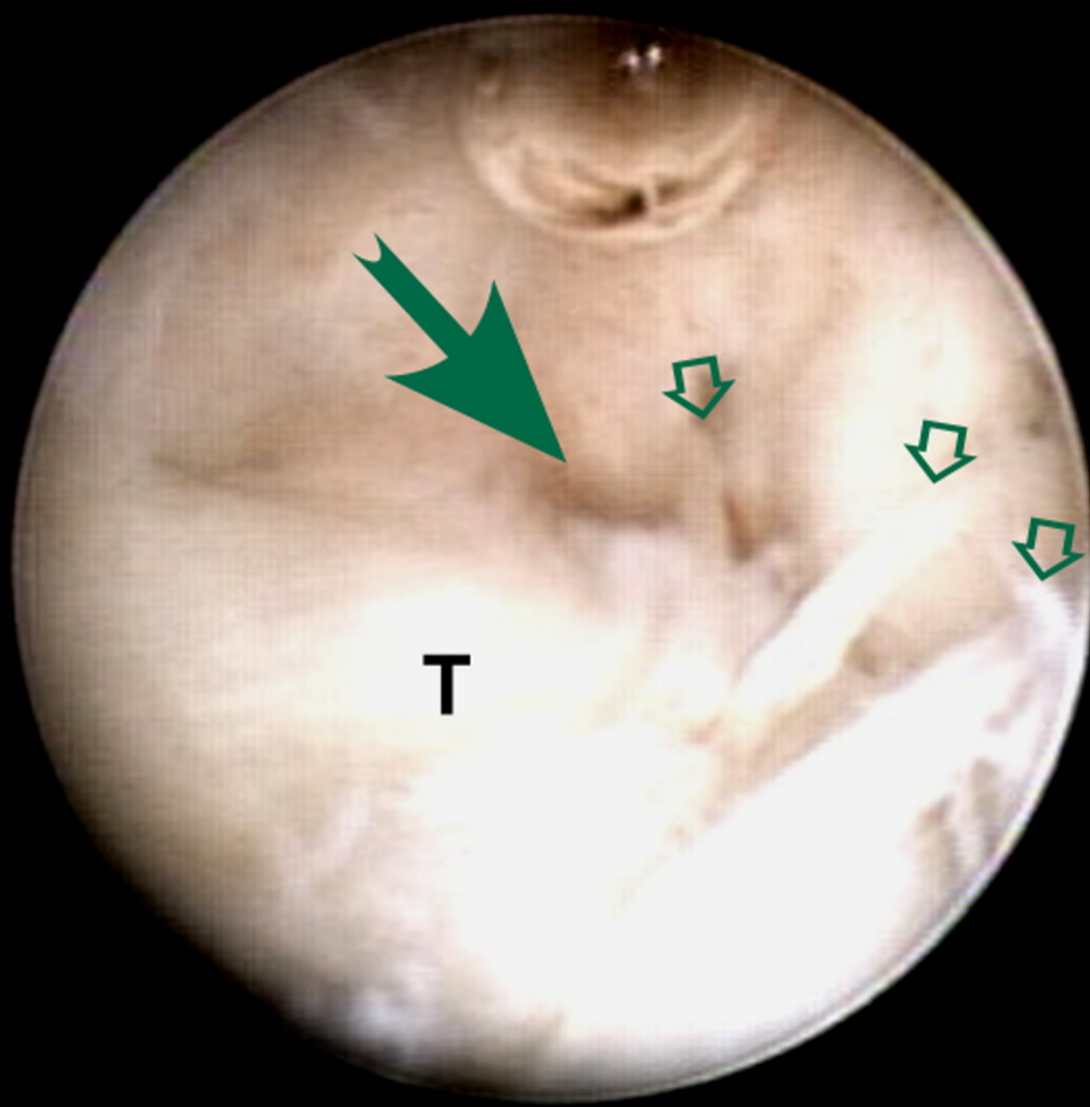
The arrow indicates the gap of TFCC (T) tear is minimized by suture repair (hollow arrows).

**Fig. (2A):**
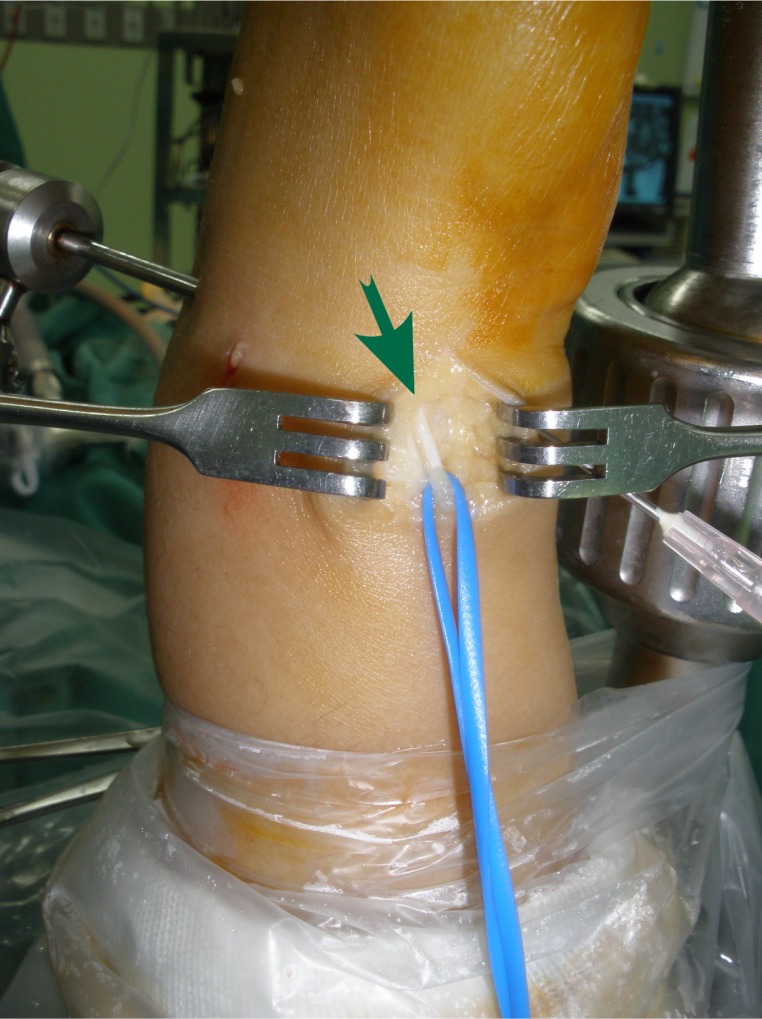
The DSBUN (arrow) is explored without soft tissue cuff.

**Fig. (2B):**
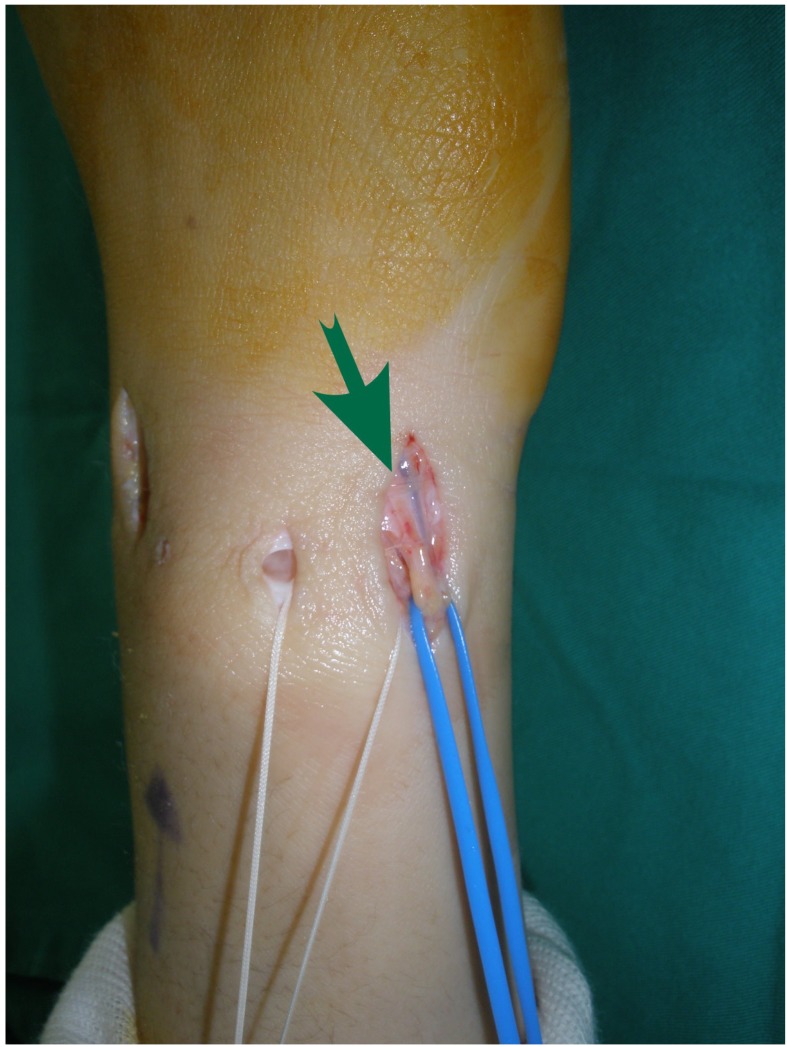
Modified perineural dissection to explore the DSBUN together with vascular tissue.

**Table 1:** Patients’ demographics.

Age (years)	34.95±10.57 (17-57)				
Sex (n=59)	Male	36 (59%)	Female		23 (41%)
Side (n=59)	Right	25 (59%)	Left	24 (41%)	
Dominant hand ^a^	Dominant	42 (71%)	Non-dominant		17 (29%)
Trauma	48 (81%)				
Sports injury	11 (19%)				
Athlete ^b^	8 (14%)				
	Racket sports: 4, Golfer: 2, Volleyball: 1, Baseball: 1				
Atzei ^c^ I	12 (20%)				
II	43 (73%)				
III	4 (7%)				
Repair method	Suture	52 (87%)	Anchor		7 (12%)
SL ligament tear ^d^	6 (10%)				
Time to OP (months) ^e^	7.22±8.43 (1-36)				
Followup (months)	23.07±15.12 (12-120)				

^a^ involving dominant or non-dominant hand

^b^ including profession athlete and school team

^c^ Atzei classification

^d^ SL = scapholunate

^e^ from injury to operation

**Table 2:** Preoperative and postoperative functional survey.

	Preoperative		Postoperative 6 months		Postoperative 1 year	
	Mean	Range	Mean	Range	Mean	Range
Mayo Score ^a^	51.44±15.57	10-70	74.32±11.50^†^	45-95	84.41±9.52^‡^	60-100
Pain	11.78±6.35	0-20	20.59±2.95^†^	15-25	22.88±2.82^‡^	20-25
Function	15.17±7.25	0-20	19.07±4.40^†^	15-25	22.2±3.12^‡^	20-25
Motion	15.76±4.24	5-20	19.32±3.41^†^	10-25	20.8±3.28 ^§^	15-25
Grip	8.39±3.4	0-15	15.34±4.99 ^†^	5-25	19.07±5.21^‡^	10-25
Grading ^b^			N = 59		N = 59	
E / G ^c^			22 (37%)		45 (76%)	
Fair			29 (49%)		13 (22%)	
Poor			8 (14%)		1 (2%)	

^a^ Mayo Modified Wrist Score (MMWS) [15].

^b^ Grading of functional outcome based on MMWS.

^c^ E = excellent; G = good.

^†^ P-value < 0.001 as compared with the preoperative data of the same item.

^‡^ P-value < 0.001 as compared to the postoperative 6-month data of the same item.

^§^ P-value = 0.02 as compared to the postoperative 6-month data of the same item.

## LIST OF ABBREVIATIONS

DSBUN: = Dorsal sensory branch of ulnar nerve
MNWS: = Mayo Modified Wrist Score
MRI: = Magnetic resonance imaging
TFCC: = Triangular fibrocartilage complex
